# Efficacy, safety, and therapeutic drug monitoring of polymyxin B sulfate and colistin sulfate in critically ill patients: a real-world retrospective study

**DOI:** 10.3389/fphar.2024.1466888

**Published:** 2025-01-03

**Authors:** Yijing Zhang, Chuhui Wang, Jiaojiao Chen, Chuqi Bai, Dan Sun, Yulan Qiu, Mengmeng Teng, Yalin Dong

**Affiliations:** ^1^ Department of Pharmacy, The First Affiliated Hospital of Xi’an Jiaotong University, Xi’an, Shaanxi, China; ^2^ Department of Pharmacy, Xi’an Hospital of Traditional Chinese Medicine, Xi’an, Shaanxi, China

**Keywords:** polymyxin B, colistin sulfate, critically ill patients, multidrug-resistant Gram-negative bacteria, therapeutic drug monitoring

## Abstract

**Background:**

Polymyxin B sulfate (PBS) and colistin sulfate (CS) are the last-line treatments for infections caused by multidrug-resistant Gram-negative bacteria, but their efficacy and safety have not been validated. The aims of the current study were to (1) determine their efficacy and safety among critically ill patients and the influencing factors, and (2) determine the relationships of drug exposure with efficacy and safety, to provide evidence for the precision dosing.

**Method:**

This retrospective study included 100 critically ill patients treated with PBS and 80 treated with CS. The efficacy outcomes were clinical efficacy and 30-day mortality, while the safety indicator was acute kidney injury (AKI) incidence.

**Result:**

There was no significant difference between the two drugs in clinical efficacy, 30-day mortality, or overall AKI incidence, but the incidence of stage 3 AKI was significantly higher in the PBS cohort than the CS cohort. Therapeutic drug monitoring (TDM) and trough concentration (C_min_) were significantly associated with clinical efficacy and AKI in both cohorts. Classification and regression tree analysis revealed that C_min_ values of ≥0.91 mg/L for PBS and C_min_ ≥ 0.53 mg/L for CS were associated with higher clinical efficacy.

**Conclusion:**

There is basically no significant difference in the efficacy and safety of PBS and CS. TDM can significantly improve the clinical efficacy of both drugs and reduce the incidence of AKI. TDM is therefore recommended to improve the clinical efficacy while reducing the adverse reactions.

## 1 Introduction

The detection rate of multidrug-resistant Gram-negative bacteria (MDR-GNB) has recently been increasing (Paul et al., 2022), making it a major threat to global public health and representing a substantial economic burden (Cassini et al., 2019; Pulingam et al., 2022). MDR-GNB infection can significantly increase the mortality risk of patients, particularly among critically ill patients (Leone et al., 2023). Due to a lack of more-effective antimicrobial drugs, polymyxins with good sensitivity to MDR-GNB have gradually become the last-line treatment (Tsuji et al., 2019; Infectious Diseases Society of China, 2021).

Polymyxin B sulfate (PBS) and colistin sulfate (CS) are two polymyxins commonly used in clinical practice (Infectious Diseases Society of China, 2021), with the latter only being available in China. The potential nephrotoxicity of polymyxins is the main factor limiting their widespread clinical use (Fiaccadori et al., 2016), with incidence rates range from 40% to 60% (Sisay et al., 2021; Wagenlehner et al., 2021). Since polymyxin-based combination therapy is the first choice for treating MDR-GNB infections (Pintado et al., 2023; Zeng et al., 2023), it is crucial to clarify the efficacy and safety of PBS and CS. However, their efficacy and safety have varied widely between studies. Some studies report that PBS is highly sensitive to MDR-GNB and has better clinical efficacy (Pan et al., 2018; Hasan et al., 2023; Jia et al., 2023), while other retrospective studies favoring the efficacy of CS with lesser nephrotoxicity (Jin et al., 2022; Lu et al., 2022; Yang et al., 2023). Besides, a recent study found no significant difference between PBS and CS in efficacy or nephrotoxicity (Wang et al., 2023), though the sample size involved in this study was relatively small. Therefore, more studies are needed urgently to assess the efficacy and safety of the two drugs.

Due to the narrow therapeutic window of polymyxins, which almost overlaps with the nephrotoxicity-concentration, and the large interindividual variability in critically ill patients, therapeutic drug monitoring (TDM) has been recommended for polymyxins (Tsuji et al., 2019; Infectious Diseases Society of China, 2021). Some studies have found that the trough concentration (C_min_) of PBS is significantly associated with clinical efficacy and acute kidney injury (AKI) incidence (Han et al., 2022; Yang et al., 2022). However, relevant studies on the exposure-response relationship in PBS are still lacking. Meanwhile, to our knowledge, there has been no investigation of the exposure–response relationship in CS to date.

We therefore conducted this retrospective study to (1) determine the efficacy and safety of PBS and CS in the treatment of critically ill patients with MDR-GNB infections and the associated factors, and (2) to determine the relationships of their concentration with efficacy and safety, to provide evidence-based support for clinical drug use in critically ill patients.

## 2 Methods

### 2.1 Clinical data collection

This single-center retrospective study included critically ill patients who received intravenous PBS or CS at the First Affiliated Hospital of Xi’an Jiaotong University from August 2021 to December 2023. The study was approved by the Ethics Committee of the First Affiliated Hospital of Xi’an Jiaotong University (XJTU1AF2024LSK-2023-432). The inclusion criteria were (1) aged ≥18 years, (2) critically ill patients receiving intravenous PBS or CS, and (3) receiving PBS or CS therapy ≥72 h. The exclusion criteria were (1) incomplete medical records and laboratory information, such as missing liver or renal function indicators on the baseline; (2) pregnant or lactating female; and (3) receiving PBS and CS simultaneously.

The clinical data were extracted retrospectively from the electronic medical record and nursing systems. The following information was collected for each patient: (1) demographic information, such as sex, age, and BMI; (2) types of infection and other underlying diseases; (3) PBS or CS regimen and concomitant medication; (4) laboratory test results during treatment; and (5) TDM results after 3 days of medication.

### 2.2 Treatment options for polymyxin B and colistin sulfate

Commonly used regimens for the injected PBS (Shanghai No. 1 Biochemical & Pharmaceutical, national drug approval number H31022631, 500,000 units/tube) in clinical practice including: 0.5 million units q12 h; 0.5 million units q8 h; 0.75 million units q12 h and 1.0 million units q12 h.

Commonly used regimens for the injected CS (Shanghai New Asiatic Pharmaceutical, national drug approval number H31020822, 500,000 units/tube) in clinical practice including: 0.5 million units q12 h; 0.75 million units q12 h and 1.0 million units q12 h. Since this was a retrospective study, the daily dose and administration times were determined by the clinicians based on actual conditions or after consultation with the clinical pharmacists.

### 2.3 Blood sample collection, analysis, and testing

After PBS or CS was administered for 3 days to achieve a steady state, C_min_ was clinically sampled 30 min before the next dose. The concentrations were measured using a validated liquid chromatography–tandem mass spectrometry method, and the mass spectrometry detection uses the triple quadrupole (Ma et al., 2022; Zhang et al., 2023). The intra- and inter-day coefficients of variation were <10%. The linear ranges of polymyxin B_1_ and polymyxin B_2_ were 0.18–14.22 μg/mL and 0.07–5.78 μg/mL, respectively, and those of polymyxin E_1_ and polymyxin E_2_ were 0.25–12.42 μg/mL and 0.15–7.58 μg/mL, respectively. The total concentrations of PBS and CS were calculated using Equations 1, 2:

$$C_{\left(B\right)}=\left[C_{\left(B1\right)}/{\text{Mol}}_{\left(B1\right)}+C_{\left(B2\right)}/{\text{Mol}}_{\left(B2\right)}\right]*\;{\text{Mol}}_{\text{avg}\left(B\right)}$$ (1)

$$C_{\left(E\right)}=\left[C_{\left(E1\right)}/{\text{Mol}}_{\left(E1\right)}+C_{\left(E2\right)}/{\text{Mol}}_{\left(E2\right)}\right]*{\text{Mol}}_{\text{avg}\left(E\right)}$$ (2)

C represents concentration; Mol represents molar mass; Mol_avg_ represents average molar mass. Mol_(B1)_ = 1,203.48 g/mol; Mol_(B2)_ = 1,189.45 g/mol; Mol_avg(B)_ = 1,189 g/mol; Mol_(E1)_ = 1,169.48 g/mol; Mol_(E2)_ = 1,155.46 g/mol; Mol_avg(E)_ = 1,164 g/mol.

### 2.4 Outcomes

The efficacy outcomes were clinical efficacy and 30-day mortality, and the safety outcome was AKI incidence (Deng et al., 2022; Zhang et al., 2024). Clinical efficacy was defined as the improvement or disappearance of clinical symptoms and signs after receiving PBS or CS for at least 3 days, including body temperature <38.0°C and improvement in at least two inflammatory markers (decreases in peripheral blood white blood cell or C-reactive protein levels of ≥30%) (Paul et al., 2018; Yang et al., 2022; Wang et al., 2023). Patients who did not meet all these criteria were classified as clinical failures.

Polymyxins-associated AKI was defined as an increase in serum creatinine (Scr) of ≥0.3 mg/dL within 48 h or to ≥1.5 times baseline within 7 days, or a urine output of <0.5 mL/kg/h for 6 h during treatment with polymyxins or within 3 days of treatment being ceased (Kellum et al., 2013). AKI can be further classified based on the Kidney Disease Improving Global Outcomes criteria. Patients with AKI stages 1, 2, and 3 were defined as having increases in Scr to 1.5–1.9, 2.0–2.9, and 3.0 times the baseline level, respectively (Kellum et al., 2013).

### 2.5 Statstical analyses

SPSS (version 19.0), R (version 4.0.2), and GraphPad Prism (version 8.0) were used for statistical analysis and graphing. Continuous variables were quantified using mean ± standard deviation (Mean ± SD) or median and interquartile-range (IQR). Categorical variables were quantified as percentages or frequencies. For differences between the two cohorts, Student’s *t* tests or nonparametric tests were used to compare continuous variables, and chi-square tests or Fisher’s exact tests were used to compare categorical variables. Logistic regression analysis was used to identify the factors associated with clinical efficacy, while the Cox proportional-hazards model was used to identify the factors associated with AKI incidence and 30-day mortality (with the time information). Data for which *P* < 0.1 in the univariate analyses were included in the multivariate logistic regression analysis or multivariate Cox proportional-hazards model. A probability value of *P* < 0.05 was considered significant in the multivariate analysis. Kaplan-Meier analysis was used to calculate the cumulative incidence rates of stage 3 AKI and 30-day mortality, which were compared using the log-rank test. The classification and regression tree (CART) model was finally analyzed to explore the efficacy and safety thresholds for PBS and CS concentrations.

Confounding variables were controlled in the efficacy and safety evaluations in the two cohorts by establishing propensity score matching (PSM) using nearest-neighbor matching to conduct a matching analysis between the PBS and CS cohorts, with a ratio of 1:1 and caliper of 0.2.

## 3 Results

### 3.1 Demographic characteristics of the patients

**FIGURE 1:**
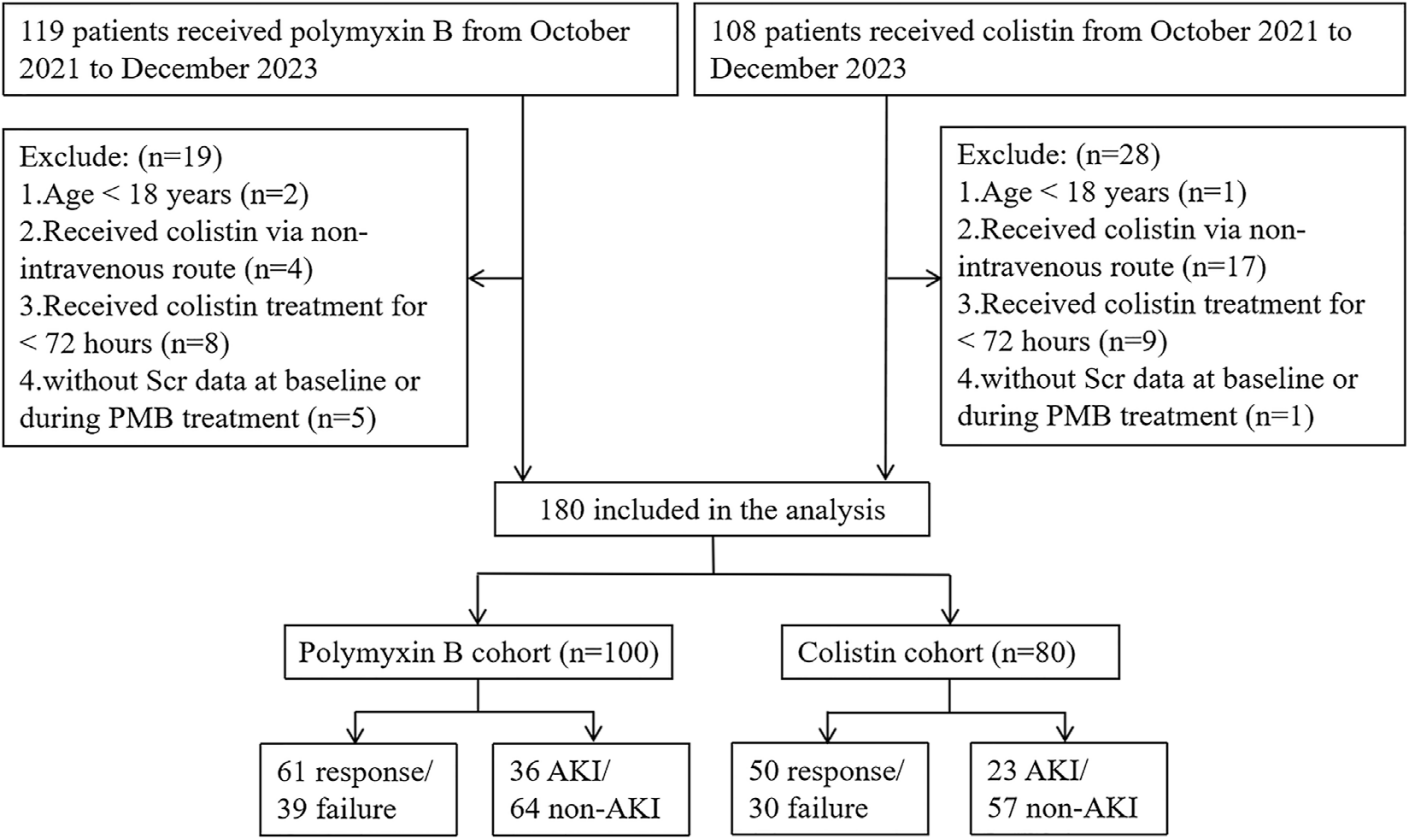
Study flow chart.

Applying the inclusion and exclusion criteria resulted in 100 and 80 patients being enrolled in the PBS and CS cohorts, respectively (Figure 1). The demographics and clinical characteristics of the patients are listed in Table 1. The median age and weight of the patients were 60 years (IQR, 50–70 years) and 67.5 kg (IQR, 59.5–76.2 kg), respectively. The most commonly presenting pathogens were carbapenem-resistant *Acinetobacter* baumannii (CRAB) (116/180, 64.4%), followed by carbapenem-resistant *Klebsiella pneumoniae* (71/180, 60.2%) and carbapenem-resistant *Pseudomonas aeruginosa* (52/180, 28.9%). Pulmonary infection was the most common type of infection (160/180, 88.9%). Among the patients treated using PBS, 61 (61/100, 61%) were treated successfully or improved, and 11 (11/100, 11.0%) eventually died; while among those treated using CS, 50 (50/80, 62.5%) were treated successfully or improved, and 12 (12/80, 15.0%) died.

**TABLE 1:** Characteristics of patients treated with polymyxin B and colistin sulfate before and after propensity score matching.

Characteristic	Before PSM			After PSM		
	Polymyxin B (n = 100)	Colistin sulfate (n = 80)	*P*	Polymyxin B (n = 59)	Colistin sulfate (n = 59)	*P*
Personal characteristics						
Age, years, median (IQR)	60.5 (52.0–71.2)	58.0 (46.5–67.0)	0.054	60 (52.0–68.0)	59 (46.0–67.0)	0.322
Sex (Male), n (%)	73 (73.0)	61 (76.3)	0.619	45 (76.3)	45 (76.3)	1
BMI, kg/m^2^, median (IQR)	22.7 (19.4–24.9)	23.0 (20.8–25.6)	0.165	22.9 (20.7–25.6)	23.5 (20.8–26.9)	0.464
hospitalization duration, days, median (IQR)	35.0 (23.0–50.5)	31.5 (21.0–43.5)	0.468	40 (24.2–54.2)	31 (21–42.5)	0.064
Length of drug treatment, days, median (IQR)	11.5 (9.0–17.0)	13.0 (8.8–22.0)	0.261	12 (9–15.8)	13 (9–22)	0.378
Baseline condition						
eGFR, mL/min·1.73 m^2^, median (IQR)	74.4 (45.7–109.2)	72.9 (35.0–104.2)	0.242	69.6 (48.4–108.1)	73.8 (36.3–101.0)	0.679
Scr, µmol/L, median (IQR)	83.5 (48.8–153.2)	89.0 (52.8–181.2)	0.214	95 (52.5–136.2)	86 (52–182)	0.684
ALT, U/L, median (IQR)	35.0 (19.0–69.2)	30.5 (15.5–75.0)	0.564	36 (18.2–72.8)	27 (14–69)	0.471
AST, U/L, median (IQR)	28.0 (21.0–46.8)	30.0 (19.8–56.5)	0.766	27 (21–46)	29 (19–54)	0.838
PCT, ng/mL, median (IQR)	1.1 (0.3–3.7)	1.5 (0.3–4.1)	0.733	1.03 (0.3–3.8)	1.25 (0.3–3.8)	0.938
Albumin, g/L, median (IQR)	32.6 (29.8–37.6)	35.1 (30.3–38.9)	0.165	31.8 (30.5–39.7)	34.1 (29.6–38.0)	0.859
Clinical conditions						
Comorbidities, n (%)						
Diabetes	29 (29.0)	15 (18.8)	0.112	22 (37.3)	13 (22.0)	0.095
Malignancy	21 (21.0)	12 (15.0)	0.367	12 (20.3)	9 (15.3)	0.470
Hypertension	36 (36.0)	35 (43.8)	0.290	22 (37.3)	28 (47.5)	0.264
Heart disease	36 (36.0)	32 (40.0)	0.452	25 (42.4)	22 (37.3)	0.573
Sepsis, n (%)	51 (51.0)	21 (26.3)	0.001	20 (33.9)	20 (33.9)	1
Sepsis stroke, n (%)	33 (33.0)	17 (21.3)	0.080	14 (23.7)	16 (27.1)	0.672
APACHE II score, median (IQR)	18.0 (13.0–23.0)	20.0 (12.8–25.5)	0.473	15.0 (12.8–22.2)	20.0 (13.0–25.0)	0.533
SOFA score, median (IQR)	8.0 (5.0–12.0)	11.0 (5.0–14.0)	0.605	9.0 (6.2–11.8)	7.0 (5.0–11.0)	0.648
Inhaled, n (%)	44 (44.0)	54 (67.5)	0.002	32 (54.2)	33 (55.9)	0.853
Surgery, n (%)	51 (51.0)	57 (71.3)	0.006	36 (61.0)	36 (61.0)	1
Infection conditions						
Type of CR-GNB, n (%)						
*Acinetobacter baumannii*	65 (65.0)	51 (63.8)	0.862	39 (66.1)	37 (62.7)	0.701
*Klebsiella pneumoniae*	38 (38.0)	33 (41.3)	0.658	24 (40.7)	25 (42.4)	0.852
*Pseudomonas aeruginosa*	32 (32.0)	20 (25.0)	0.303	16 (27.1)	16 (27.1)	1
*Escherichia coli*	8 (8.0)	4 (5.0)	0.423	2 (3.4)	3 (5.1)	1
*Other bacteria*	19 (19.0)	10 (12.5)	0.227	11 (18.6)	10 (16.9)	0.810
Multi-CR-GNB infection (≥2), n (%)	47 (47.0)	29 (36.3)	0.147	28 (47.5)	24 (40.7)	0.458
Infection site, n (%)						
Respiratory tract	90 (90.0)	70 (87.5)	0.596	54 (91.5)	49 (83.1)	0.167
Bloodstream	26 (26.0)	9 (11.3)	0.013	8 (13.6)	9 (15.3)	0.793
Abdomen	15 (15.0)	8 (10.0)	0.318	6 (10.2)	7 (11.9)	0.769
Brain	2 (2.0)	5 (6.3)	0.281	1 (1.7)	5 (8.5)	0.209
Urinary tract	10 (10.0)	10 (12.5)	0.596	7 (11.9)	9 (15.3)	0.591
Other	10 (10.0)	5 (6.3)	0.366	6 (10.2)	4 (6.8)	0.509
Multi-site infection (≥2), n (%)	38 (38.0)	25 (31.3)	0.345	19 (32.2)	22 (37.3)	0.562
In-hospital treatments						
CRRT, n (%)	40 (40.0)	39 (48.8)	0.240	24 (40.7)	28 (47.5)	0.458
TDM practice, n (%)	70 (70.0)	34 (42.5)	0.001	48 (81.4)	33 (55.9)	0.003
Concomitant antibiotics, n (%)						
Sulbactam	29 (29.0)	9 (11.3)	0.004	18 (30.5)	8 (13.6)	0.050
Ceftazidime avibactam	20 (20.0)	11 (13.8)	0.270	9 (15.3)	7 (11.9)	0.591
Cefoperazone sulbactam	20 (20.0)	10 (12.5)	0.180	13 (22.0)	8 (13.6)	0.229
Other cephalosporins	18 (18.0)	17 (21.3)	0.584	14 (23.7)	12 (20.3)	0.657
Meropenem	46 (46.0)	46 (57.5)	0.125	31 (52.5)	34 (57.6)	0.579
Other carbapenem	32 (32.0)	42 (52.5)	0.005	19 (32.2)	27 (45.8)	0.131
Amikacin	15 (15.0)	12 (15.0)	1.000	12 (20.3)	10 (16.9)	0.636
Vancomycin	52 (52.0)	35 (43.8)	0.271	31 (52.5)	32 (54.2)	0.854
Sulfamethoxazole/Trimethoprim	17 (17.0)	25 (31.3)	0.025	6 (10.2)	18 (30.5)	0.042
Omadacycline	16 (16.0)	6 (7.5)	0.084	12 (20.3)	2 (3.4)	0.050
Tigecycline	26 (26.0)	29 (36.3)	0.138	13 (22.0)	26 (44.1)	0.062
Minocycline	25 (25.0)	7 (8.8)	0.005	16 (27.1)	7 (11.9)	0.036
Amphotericin B	25 (25.0)	45 (56.2)	0.001	17 (28.8)	33 (55.9)	0.028
Acyclovir/Valacyclovir	36 (36.4)	19 (24.1)	0.077	19 (32.2)	13 (22.0)	0.213
NSAIDs	15 (15.0)	6 (7.5)	0.119	11 (18.6)	5 (8.5)	0.107
Immunosuppressant	18 (18.0)	15 (18.8)	0.897	13 (22.0)	12 (20.3)	0.822
Glucocorticoid	40 (40.0)	51 (63.8)	0.002	27 (45.8)	45 (76.3)	0.024
Antifungal drugs	74 (74.0)	51 (63.8)	0.138	42 (71.2)	39 (66.1)	0.552
Diuretics	20 (20.0)	7 (9.0)	0.042	12 (20.3)	4 (6.8)	0.065
Concurrent nephrotoxin≥2, n (%)	67 (67.0)	50 (62.5)	0.529	39 (66.1)	40 (67.8)	0.845

Abbreviations: PSM, propensity score matching; IQR, interquartile range; BMI, body mass index; eGFR, estimated glomerular filtration rate; Scr, serum creatinine; ALT, alanine aminotransferase; AST, aspartate aminotransferase; PCT, procalcitonin; APACHE, acute physiology and chronic health evaluation; SOFA, sequential organ failure assessment; CR-GNB, Carbapenem resistant gram-negative bacteria; CRRT, continuous renal replacement therapy; TDM, therapeutic drug monitoring; NSAIDs, Nonsteroidal Anti-inflammatory Drugs.

### 3.2 Efficacy and safety analysis of polymyxin B and colistin sulfate

After PSM, 118 patients (59 patients each) who received PBS or CS were matched, for which the demographics and clinical characteristics are listed in Table 1. The data of the two cohorts after matching were balanced in terms of weight, age, infection type, comorbidities, and laboratory test results, with no significant differences being identified.

**FIGURE 2:**
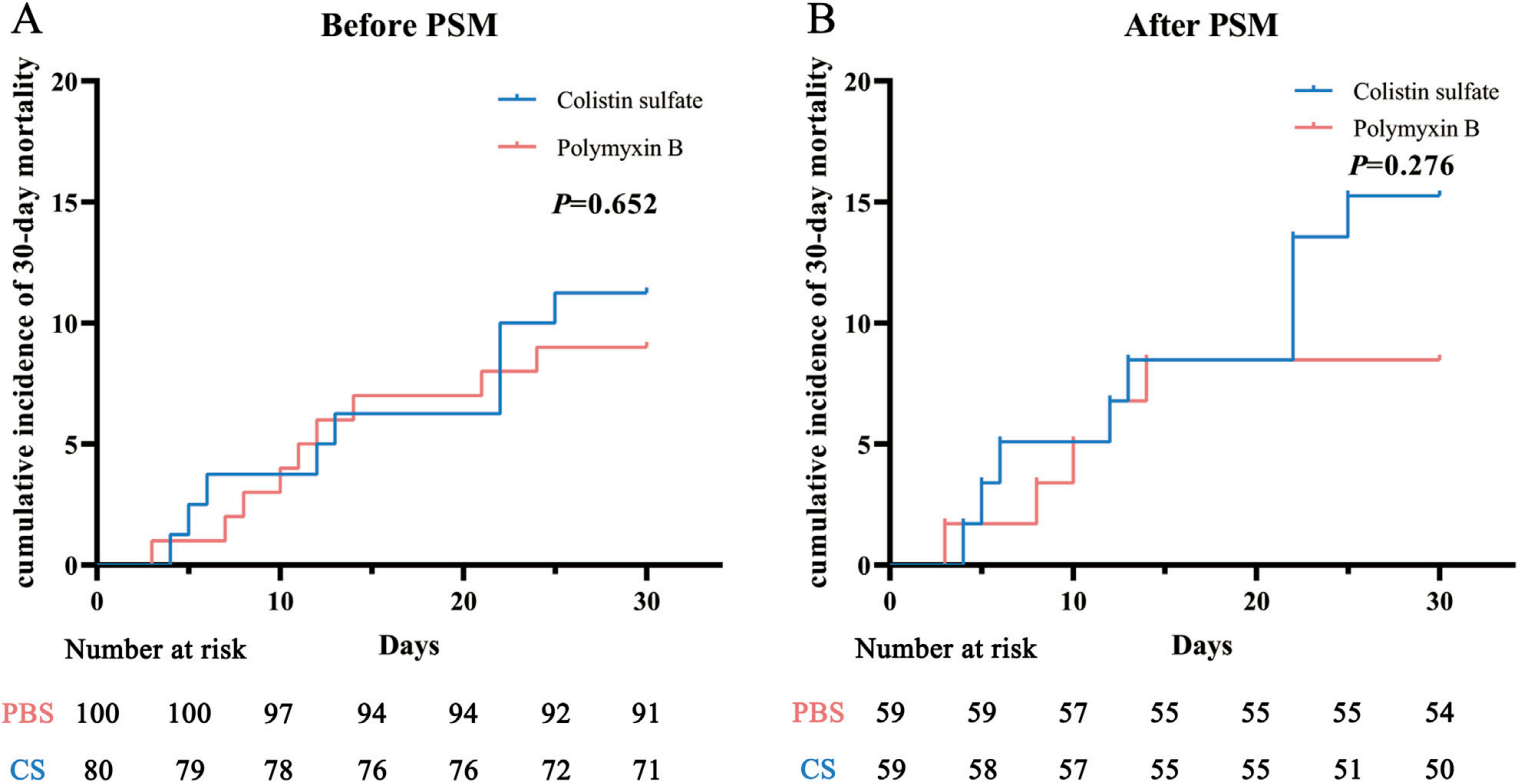
Cumulative incidence of 30-day mortality before **(A)** and after **(B)** propensity score matching (PSM) in the polymyxin B and colistin sulfate cohorts. Abbreviations: PSM, propensity score matching.

There were no significant differences in the clinical efficacies of PBS and CS before or after PSM (*P* = 0.837 and *P* = 0.566). The clinical efficacy rates of PBS and CS before PSM were 61.0% and 62.5%, respectively; after PSM these were 61.0% and 66.1%. Significant differences were also not observed in 30-day mortality between the two drugs before and after PSM (*P* = 0.633 and *P* = 0.255). The 30-day mortality rates of PBS and CS before PSM were 9.0% and 11.2%, respectively; after PSM these were 8.5% and 15.2%. Kaplan-Meier curve analysis (Figure 2) also indicated that there were no significant differences between the two cohorts (*P* = 0.652 and *P* = 0.276).

**FIGURE 3:**
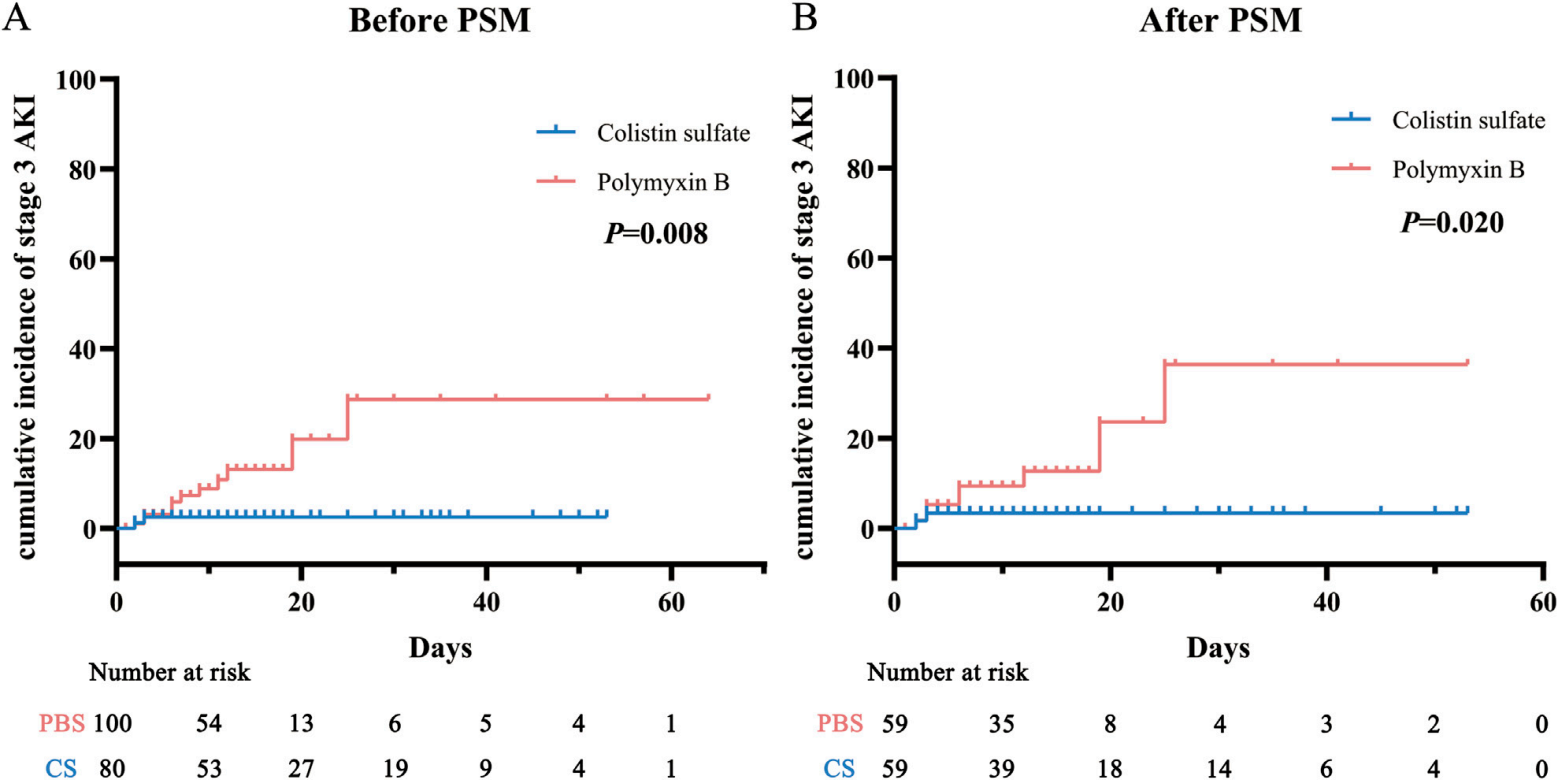
Cumulative incidence of stage 3 acute kidney injury (AKI) before **(A)** and after **(B)** propensity score matching (PSM) in the polymyxin B and colistin sulfate cohorts. Abbreviations: PSM, propensity score matching; AKI, acute kidney injury.

AKI incidence rates in each stage were analyzed before and after matching (Table 2): they were 36.0% and 28.8% for PBS and CS, respectively, before PSM, and 28.8% and 30.5% after PSM. The chi-square tests indicated that there was no significant difference in all-stage AKI incidence between the two cohorts (*P* = 0.303 and *P* = 0.840), but for stage 3 AKI, the incidence of PBS was significantly higher than that of CS (*P* = 0.029 and *P* = 0.047). The Kaplan-Meier curve analysis (Figure 3) also obtained the same results.

**TABLE 2:** The incidence of acute kidney injury (AKI) in patients treated with polymyxin B and colistin sulfate.

Outcomes	Before PSM			After PSM		
	Polymyxin B (n = 100)	Colistin sulfate (n = 80)	*P*	Polymyxin B (n = 59)	Colistin sulfate (n = 59)	*P*
AKI, n (%)						
All stage	36 (36.0)	23 (28.8)	0.303	17 (28.8)	18 (30.5)	0.840
Stage 1	15 (15.0)	11 (13.8)		5 (8.5)	8 (13.6)	
Stage 2	10 (10.0)	10 (12.5)		4 (6.8)	8 (13.6)	
Stage 3	11 (11.0)	2 (2.5)	**0.029**	8 (10.2)	2 (3.4)	**0.047**

Abbreviations: PSM, propensity score matching; AKI, acute kidney injury. Bold values indicate *P* < 0.05.

### 3.3 Factors associated with efficacy and safety

#### 3.3.1 Factors associated with the efficacy and safety of polymyxin B

The univariate analyses (Table 3) indicated that TDM practice, albumin, acute physiology and chronic health evaluation (APACHE) II score, sequential organ failure assessment (SOFA) score, CRAB infection, length of using drugs, and the concomitant use of meropenem, omadacycline, or cefoperazone-sulbactam were associated with clinical efficacy. Factors for which *P* < 0.1 in the univariate analyses were included in the multivariate analysis. TDM practice (*P* = 0.020), lower APACHE II score (*P* = 0.044), and concomitant meropenem use (*P* = 0.014) were significantly related to better clinical efficacy in the multivariate analysis. We also found that procalcitonin (*P* = 0.024), APACHE II score (*P* = 0.003), concurrent malignancy (*P* = 0.034), and septic shock (*P* = 0.044) were significantly associated with 30-day mortality (Supplementary Table S1).

**TABLE 3:** Univariate and multivariate analysis for clinical efficacy and acute kidney injury of polymyxin B.

Clinical efficacy						
Variables	Univariate analysis			Multivariate analysis		
	OR	95%CI	*P*	OR	95%CI	*P*
TDM practice	3.155	1.291–7.714	0.012	7.557	1.379–41.418	**0.020**
APACHE II	0.888	0.820–0.961	0.003	0.886	0.788–0.997	**0.044**
SOFA	0.894	0.806–0.991	0.033			
Albumin	1.081	1.004–1.164	0.040			
Length of using drug, days	1.077	1.014–1.144	0.016			
*Acinetobacter baumannii*	0.404	0.164–0.995	0.049			
Meropenem	2.833	1.214–6.610	0.016	10.438	1.714–11.578	**0.011**
Omadacycline	3.250	0.862–12.260	0.082			
Cefoperazone/Sulbactam	0.340	0.124–0.930	0.036			

Acute kidney injury						
Variables	HR	95%CI	*P*	HR	95%CI	*P*
TDM practice	0.396	0.197–0.797	0.009	0.285	0.089–0.913	**0.035**
Length of hospital stay, days	0.983	0.970–0.997	0.014			
APACHE II	1.071	1.018–1.126	0.008	1.039	1.002–1.102	**0.034**
SOFA	1.070	0.996–1.150	0.063			
Malignancy	2.295	1.116–4.719	0.084			
Sepsis	3.178	1.494–6.760	0.003	5.639	1.433–22.184	**0.013**
Sepsis stroke	1.851	0.960–3.570	0.066			
Meropenem	0.544	0.275–1.077	0.081			
Sulfamethoxazole/Trimethoprim	0.479	0.208–1.104	0.024	3.163	1.171–8.549	**0.023**

Abbreviations: OR, odds ratio; HR, hazard ratio; CI, confidence interval; APACHE, acute physiology and chronic health evaluation; SOFA, sequential organ failure assessment; TDM, therapeutic drug monitoring. Bold values indicate *P* < 0.05.

Univariate analyses (Table 3) were also performed to identify the risk factors associated with AKI, which found that TDM practice, hospitalization duration, APACHE II score, SOFA score, sepsis, septic shock, concurrent neoplastic disease, and concomitant use of meropenem or sulfamethoxazole-trimethoprim (TMP-SMZ) were associated with AKI. In the multivariate analysis, TDM practice (*P* = 0.035), sepsis (*P* = 0.013), APACHE II score (*P* = 0.034), and concomitant sulfamethoxazole/trimethoprim (TMP-SMZ) (*P* = 0.023) were associated with AKI risk for those treated using PBS.

#### 3.3.2 Factors associated with the efficacy and safety of colistin sulfate

Univariate analyses were performed to identify the factors significantly influencing the clinical efficacy of CS (Table 4). The results showed that TDM practice, aspartate aminotransferase, APACHE II score, SOFA score, concurrent heart disease, infection with at least two organisms, and concomitant use of cefoperazone-sulbactam or other cephalosporins were significant correlation with clinical efficacy. In multivariate analysis, TDM practice (*P* = 0.047), APACHE II score (*P* = 0.048), and infection with ≥2 organisms (*P* = 0.008) were significantly associated with clinical efficacy. We also found that APACHE II score (*P* = 0.008), concurrent diabetes (*P* = 0.031), and septic shock (*P* = 0.017) were significantly associated with 30-day mortality (Supplementary Table S2).

**TABLE 4:** Univariate and multivariate analysis for clinical efficacy and acute kidney injury of colistin sulfate.

Clinical efficacy						
Variables	Univariate analysis			Multivariate analysis		
	OR	95%CI	*P*	OR	95%CI	*P*
TDM practice	2.979	1.117–7.947	0.029	5.323	1.025–27.641	**0.047**
AST, U/L	0.991	0.981–1.001	0.087			
Heart disease	0.412	0.162–1.045	0.062			
Multi-CR-GNB infection (≥2)	0.241	0.091–0.638	0.004	0.159	0.041–0.623	**0.008**
Cefoperazone/Sulbactam	6.366	0.764–53.035	0.087			
Other Carbapenem	0.326	0.108–0.980	0.046			
APACHE II	0.810	0.726–0.905	0.001	0.867	0.753–0.999	**0.048**
SOFA	0.864	0.769–0.972	0.015			

Acute kidney injury						
Variables	HR	95%CI	*P*	HR	95%CI	*P*
TDM practice	0.311	0.104–0.093	0.037	0.275	0.077–0.988	**0.048**
Scr, µmol/L	1.005	1.001–1.009	0.007	1.006	1.002–1.010	**0.032**
ALT, U/L	1.008	1.000–1.018	0.072			
Amikacin	5.679	2.242–14.383	0.001	3.076	1.041–9.092	**0.042**
Cefoperazone/Sulbactam	4.103	1.630–10.331	0.003			
Sulfamethoxazole/Trimethoprim	3.069	1.270–7.413	0.013			

Abbreviations: OR, odds ratio; HR, hazard ratio; CI, confidence interval; Scr, serum creatinine; ALT, alanine aminotransferase; AST, aspartate aminotransferase; APACHE, acute physiology and chronic health evaluation; SOFA, sequential organ failure assessment; CR-GNB, Carbapenem resistant gram-negative bacteria; TDM, therapeutic drug monitoring. Bold values indicate *P* < 0.05.

Univariate analyses identified that the risk factors associated with AKI were TDM practice, Scr, alanine aminotransferase values, and the concomitant use of amikacin, cefoperazone-sulbactam, or TMP-SMZ. In the multivariate analysis, TDM practice (*P* = 0.048), creatinine (*P* = 0.007), and concomitant amikacin use (*P* = 0.042) were associated with AKI risk for those treated using CS (Table 4).

### 3.4 Relationships of polymyxin B and colistin sulfate concentrations with efficacy and safety

TDM has a significant impact on the clinical efficacy and AKI incidence in PBS and CS. The C_min_ of PBS was notably demonstrated to be significantly related to the area under the plasma concentration-time curve (Yang et al., 2022), while the C_min_ of colistin has also been recommended as an indicator for TDM (Abdul-Aziz et al., 2020). Subgroup analysis was therefore applied to 70 out of 100 patients with PBS (106 C_min_) who received TDM, and 34 out of 80 patients with CS (53 C_min_) who received TDM. PBS and CS treatments were effective in 45 and 26 patients, respectively, and 23 and 11 patients developed AKI. The results indicated that the C_min_ values of PBS and CS were significantly correlated with the clinical efficacy of the drugs and AKI incidence (*P* = 0.007 and *P* = 0.026; *P* = 0.008 and *P* = 0.037). The clinical efficacy and AKI incidence will also increase with the concentration increases.

**FIGURE 4:**
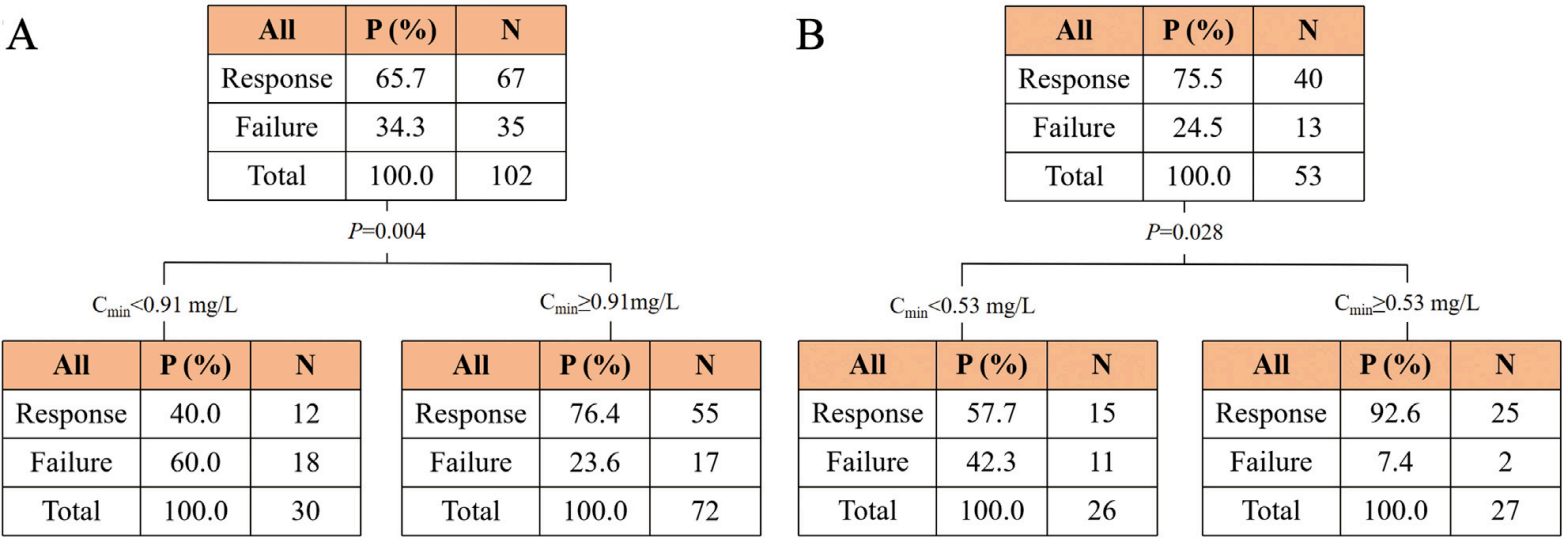
Classification and regression tree results for the incidence of clinical efficacy in the polymyxin B **(A)** and colistin sulfate **(B)** cohorts. Abbreviations: C_min_, trough concentration.

Moreover, a CART model was analyzed to predict the clinical efficacy and safety thresholds of C_min_ for PBS and CS (Figure 4). The clinical efficacy of PBS and CS were significantly increased at C_min_ values of ≥0.91 mg/L and ≥0.53 mg/L, respectively. The threshold for nephrotoxicity was not established.

The concentrations of PBS and CS were divided into two groups based on the thresholds, respectively (Supplementary Table S3). The chi-square test revealed a significant increase in efficacy for PBS or CS when the concentration was higher than the cutoff value (*P* < 0.001 and *P* = 0.001).

## 4 Discussion

The emergence of MDR-GNB has become a global public health concern, and polymyxins have become one of the most important treatment options for MDR-GNB due to their unique mechanism of action (Nang et al., 2021). After using PSM to ensure that the baseline characteristics of the two cohorts were balanced and comparable, this study analyzed the clinical efficacy and nephrotoxicity of critically ill patients treated with PBS and CS for MDR-GNB infections. And the study also explored the correlations among concentration, clinical efficacy, and AKI incidence.

This study included 100 patients treated with PBS and 80 patients treated with CS. The most commonly treated pathogen was CRAB, and the most common infection site was the respiratory tract. The results indicated that there was no significant difference in clinical efficacy and 30-day mortality between PBS and CS in treating MDR-GNB infection, which was consistent with the findings of Wang et al. (2023). There was no significant difference in the incidence of all-stage AKI between the two drugs, but stage 3 AKI had a significantly higher incidence in PBS than in CS. This result was also similar to those of Zhang et al. (2024). Although this study found that the incidence of stage 3 AKI was higher with PBS compared to CS, the number of patients who developed stage 3 AKI was relatively small, accounting for 11% (11 cases) and 2.5% (2 cases) of the total number, respectively. Further research with a larger sample size was needed in the future. The widespread issue of nephrotoxicity with polymyxins necessitates routine kidney function monitoring and TDM in clinical practice, especially for patients with pre-existing renal impairment or other risk factors. This approach helps detect and manage AKI early, preventing the condition from worsening.

This study further explored the factors associated with clinical efficacy and AKI incidence in PBS and CS. We found that TDM practice can significantly improve the clinical efficacy and AKI incidence in PBS and CS, indicating that timely TDM and appropriately adjusted regimens are necessary for future clinical practice, and can help to improve the clinical efficacy and reduce the incidence of adverse reactions. The APACHE II score was also significantly correlated with the clinical efficacy of both drugs, providing clear quantitative data regarding efficacy, with higher scores suggesting more-severe disease and a higher risk of treatment failure. Additionally, the study found that concurrently using meropenem significantly increased the clinical efficacy of PBS. The potential advantage of this combination treatment is the synergistic effects, which may improve efficacy and prevent the development of drug resistance (Ni et al., 2015). The analysis of risk factors associated with AKI incidence revealed that concurrently using TMP-SMZ and amikacin significantly increases the AKI risk for PBS and CS, respectively. Since both amikacin and TMP-SMZ are cleared through the kidneys (Li et al., 2020) and amikacin has significant nephrotoxicity (Chan et al., 2020), combining them with polymyxins may increase the burden on the kidneys and increase the AKI risk. Since polymyxin-based combinations are often used to clinically treat MDR-GNB infections, and The European Committee on Antimicrobial Susceptibility Testing suggests that polymyxins should not be used as monotherapy, except when high exposure could be achieved at the infection site (EUCAST, 2021), exploring better combination options remains a major challenge in treating patients with MDR-GNB infection. Various factors should be considered when performing combination therapy, especially if the patient possesses other risk factors for nephrotoxicity. Meanwhile, higher Scr levels was associated with a higher risk of AKI in CS, suggesting that we should also closely monitor the renal function of patients during clinical treatment.

The results found that TDM practice could significantly reduce the AKI incidence in PBS and CS, but it did not affect 30-day mortality rates, and this result was similar with Li et al. (2024). This may be because that the included patients were all critically ill patients. Although TDM increased the efficacy of polymyxins in treating Gram-negative bacterial infections and reduced the incidence of AKI, the mortality rate of critically ill patients is affected by multiple factors, including the severity of underlying illness, comorbidities, and overall organ function. Therefore, no significant impact of TDM on 30-day all-cause mortality was observed.

In this study we also explored the correlations of C_min_ with the clinical efficacy and safety of the two drugs. We found that higher C_min_ values of PBS and CS were significantly associated with enhanced clinical efficacy and increased AKI incidence. This result confirmed the feasibility of adjusting the dosage regimen based on C_min_ values while performing TDM in clinical practice. The CART analysis indicated significant improvements in clinical efficacy at C_min_ values of ≥0.91 mg/L and ≥0.53 mg/L for PBS and CS, respectively. This was close to the efficacy threshold of C_min_ ≥1.01 mg/L for PBS identified by Yang et al. (2022). Moreover, we established a novel efficacy threshold for CS for the first time. More large-sample multicenter studies are needed to further validate these results.

This study also had some limitations. Firstly, it was a single-center retrospective design. Although the patient baseline values were balanced using PSM, this design may have inherently introduced bias. Prospective studies are therefore needed to validate the results of this study. Secondly, the samples of concentration included in the TDM analysis were limited, which precluded us from establishing a safety threshold. Thirdly, since MDR-GNB infections are often treated using polymyxin-based combination therapy and nephrotoxic drugs were sometimes used in combination, it was challenging to assess the clinical efficacy and safety of polymyxins independently.

## 5 Conclusion

PBS and CS can both be used to treat MDR-GNB infection in critically ill patients. This study found no significant difference in clinical efficacy and all-stage AKI incidence between PBS and CS, while stage 3 AKI had a significantly lower incidence in CS than in PBS. Moreover, the C_min_ values of the two drugs were associated with clinical efficacy and AKI incidence. Clinical efficacy was significantly increased at C_min_ values of ≥0.91 mg/L for PBS and ≥0.53 mg/L for CS. Future multicenter studies with larger samples are needed to verify these results. TDM needs to be applied in clinical practice to improve the clinical cure rate and reduce the occurrence of adverse reactions.

## Data Availability

The original contributions presented in the study are included in the article/Supplementary Material, further inquiries can be directed to the corresponding author.
