# Supplementary material for: Efficacy, safety, and therapeutic drug monitoring of polymyxin B sulfate and colistin sulfate in critically ill patients: a real-world retrospective study
Source: Front Pharmacol. 2025 Jan 3;15:1466888. doi: 10.3389/fphar.2024.1466888 (PMC11739331; doi:10.3389/fphar.2024.1466888)
Supplement: Supplementary file 1 [file Table1.docx]

Supplementary Material

**Supplementary Table S1** Univariate and multivariate analysis for 30-day motaility of polymyxin B.

|  | **Univariate Analysis** | | | **Multivariate Analysis** | | |
| --- | --- | --- | --- | --- | --- | --- |
| **Variable** | **HR** | **95%CI** | ***P*** | **HR** | **95%CI** | ***P*** |
| AST | 1.001 | 1.000-1.001 | 0.083 |  |  |  |
| PCT | 1.021 | 1.002-1.040 | 0.033 | 1.028 | 1.004-1.052 | **0.024** |
| Albumin | 0.821 | 0.702-0.961 | 0.014 |  |  |  |
| APACHE II | 1.105 | 1.017-1.201 | 0.019 | 1.134 | 1.045-1.230 | **0.003** |
| SOFA | 1.166 | 1.013-1.342 | 0.033 |  |  |  |
| Malignancy | 5.536 | 1.485-20.632 | 0.011 | 4.736 | 1.123-19.982 | **0.034** |
| Sepsis | 8.546 | 1.068-68.348 | 0.043 |  |  |  |
| Sepsis stroke | 19.290 | 2.410-154.363 | 0.005 | 10.260 | 1.064-98.911 | **0.044** |

Abbreviations: HR, hazard ratio; CI, confidence interval; AST, aspartate aminotransferase; PCT, procalcitonin; APACHE, Acute Physiology and Chronic Health Evaluation; SOFA, Sequential Organ Failure Assessment. Bold values indicate *P* <0.05.

**Supplementary Table S2** Univariate and multivariate analysis for 30-day motaility of colistin sulfate.

|  | **Univariate Analysis** | | | **Multivariate Analysis** | | |
| --- | --- | --- | --- | --- | --- | --- |
| **Variable** | **HR** | **95%CI** | ***P*** | **HR** | **95%CI** | ***P*** |
| Length of hospital stay | 0.949 | 0.895-1.005 | 0.074 |  |  |  |
| Diabetes | 5.941 | 1.593-22.159 | 0.008 | 4.298 | 1.139-16.222 | **0.031** |
| APACHE II | 1.140 | 1.040-1.249 | 0.005 | 1.237 | 1.058-1.447 | **0.008** |
| SOFA | 1.258 | 1.095-1.446 | 0.001 |  |  |  |
| Multi-CR-GNB infection (≥2) | 3.857 | 0.964-15.429 | 0.056 |  |  |  |
| Sepsis stroke | 7.312 | 1.828-29.259 | 0.005 | 5.481 | 1.360-22.084 | **0.017** |

Abbreviations: HR, hazard ratio; CI, confidence interval; APACHE, Acute Physiology and Chronic Health Evaluation; SOFA, Sequential Organ Failure Assessment; CR-GNB, Carbapenem resistant Gram-negative bacteria. Bold values indicate *P* <0.05.

**Supplementary Table S3** Chi-square test of concentration and clinical efficacy of polymyxin B and colistin sulfate.

| **Polymyxin B** | |  |  |
| --- | --- | --- | --- |
| **Variable** | **C_min_<0.91 mg/L (n=31)** | **C_min_≥0.91 mg/L (n=75)** | ***P*** |
| Response | 11 (35.5%) | 58 (77.3%) | **<0.001** |
| **Colistin Sulfate** | |  |  |
| **Variable** | **C_min_<0.53 mg/L (n=30)** | **C_min_≥0.53 mg/L (n=23)** | ***P*** |
| Response | 19 (63.3%) | 22 (95.6%) | **0.001** |

Abbreviations: C_min_, trough concentration. Bold values indicate *P* <0.05.
